# Infantile-onset Alexander disease in a child with long-term follow-up by serial magnetic resonance imaging: a case report

**DOI:** 10.1186/1752-1947-7-194

**Published:** 2013-07-26

**Authors:** Fumiko Nishibayashi, Miho Kawashima, Yoshiaki Katada, Nobuyuki Murakami, Miwako Nozaki

**Affiliations:** 1 Department of Radiology, Dokkyo Medical University Koshigaya Hospital, 2-1-50, Minamikoshigaya, Koshigaya-shi, Saitama 343-8555, Japan; 2 Department of Pediatrics, Dokkyo Medical University Koshigaya Hospital, 2-1-50, Minamikoshigaya, Koshigaya-shi, Saitama 343-8555, Japan

## Abstract

**Introduction:**

Alexander disease is a rare disorder resulting from a glial fibrillary acidic protein gene mutation which causes progressive degeneration of white matter. With the usual poor prognosis, there are few case reports with long-term follow-up. We report the five-year clinical course of Alexander disease in one case using serial magnetic resonance imaging.

**Case presentation:**

A 12-month-old Japanese male was referred to the pediatrics department in our hospital because of developmental retardation. Alexander disease was diagnosed by gene examination of the mutation of a glial fibrillary acidic protein. Magnetic resonance imaging findings showed abnormalities in white matter, deep gray matter, and medulla oblongata. Serial magnetic resonance imaging examinations until the age of five were performed and changes in magnetic resonance imaging findings were compared to the progression in clinical symptoms.

**Conclusion:**

Alexander disease is a very rare disease with a variety of clinical phenotypes. Therefore serial magnetic resonance imaging studies for long-term survival infantile cases including our case may be important in the analysis of the pathophysiological mechanism.

## Introduction

Alexander disease is a rare disorder resulting from a glial fibrillary acidic protein (GFAP) gene mutation which causes progressive degeneration in white matter. Most cases are solitary, but hereditary cases have also been reported. Abnormalities predominate in the white matter of both frontal lobes, making magnetic resonance imaging (MRI) useful for diagnosis. In this report, we show the clinical course of Alexander disease in one case using MRI.

## Case presentation

A 12-month-old Japanese male was referred to the pediatrics department of our hospital because he was unable to sit independently. At the initial examination, the 12-month-old could hold his head up but could not maintain a sitting posture. Macrocephaly was not noted. He had no history of perinatal abnormalities. Birth weight was 3058g, height 49cm, and head circumference 34.5cm. There was no family history of neurological disorder.

**Figure 1:**
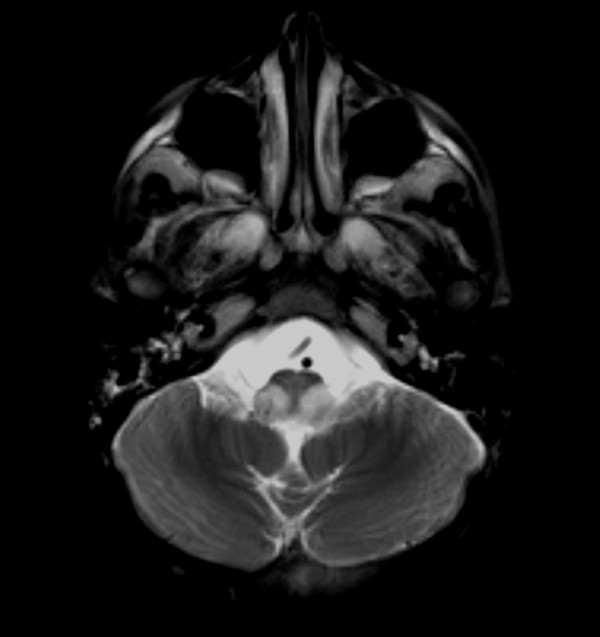
**Brain computed tomography and magnetic resonance imaging at 12 months. a** Computed tomography image demonstrates low attenuation areas in the white matter of the frontal lobes, putamen, external capsule and claustrum, and cystic formation in the white matter of the frontal lobes. **b** T2-weighted image shows areas of high signal intensity and cystic formation in the white matter of the frontal lobes, and significant deep gray matter structure abnormality. **c** T2-weighted image shows small, bilateral hyperintense lesions in the medulla oblongata. **d** Contrast T1-weighted image demonstrates contrast enhancement in the pericystic lesion and the caudate nucleus.

Brain computed tomography (CT) showed areas of low attenuation with frontal white matter preponderance, cyst formation near the anterior horn of the lateral ventricle, and cerebral atrophy predominant in the frontal lobes (Figure 1a). T2-weighted images demonstrated symmetrical high-intensity areas in the white matter of both frontal lobes and deep gray matter, cyst formation in the white matter, and cerebral atrophy (Figure 1b). T2-weighted images also showed slight abnormalities in the medulla oblongata (Figure 1c). Contrast-enhanced T1-weighted images showed contrast enhancement of the peri-cystic area and caudate nucleus region bilaterally (Figure 1d).

These findings suggested infantile leukodystrophy, and other diseases in the differential diagnosis including Alexander disease, megalencephalic leukoencephalopathy with subcortical cysts (MLC), van der Knaap disease, Canavan disease, Pelizaeus-Merzbacher disease, and metachromatic leukodystrophy. Alexander disease or MLC were strongly suspected, as these types of infantile leukodystrophy involve cyst formation. Alexander disease was diagnosed by gene examination, and Murakami *et al*. already reported by the mutation of the *GFAP* gene in this case: the patient had a heterozygous deletion of genomic sequence 1247-1249GGG>GG in exon 8 of the *GFAP* gene [1].

**Figure 2:**
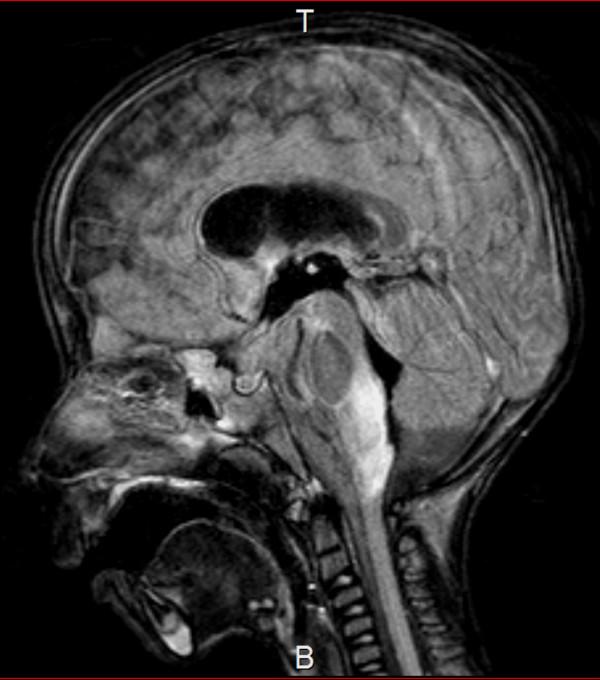
**Magnetic resonance imaging at 4 years. a** T2-weighted image shows significant volume loss in bilateral white matter and deep gray matter as a cause of enlargement of the anterior horn of the lateral ventricle. **b1,2** T1-weighted image and T2-weighted image show the periventricular rim around the posterior horn of the lateral ventricle. **c** Abnormal signals in the medulla oblongata become apparent in T2-weighted image.

This case was followed with the best supportive care and careful rehabilitation for almost 5 years after the onset of the disease. His general condition was stable. The second MRI was performed at 4 years of age after his spastic quadriplegia began to slowly progress. Significant volume loss in bilateral white matter and deep gray matter as a cause of enlargement of the anterior horn of the lateral ventricle was recognized from T2-weighted images (Figure 2a). Furthermore, a periventricular rim of high signal intensity on T1-weighted images and low signal intensity on T2-weighted images was apparent (Figure 2b), and high intensity lesions on T2-weighted images became more apparent in the medulla oblongata (Figure 2c).

**Figure 3:**
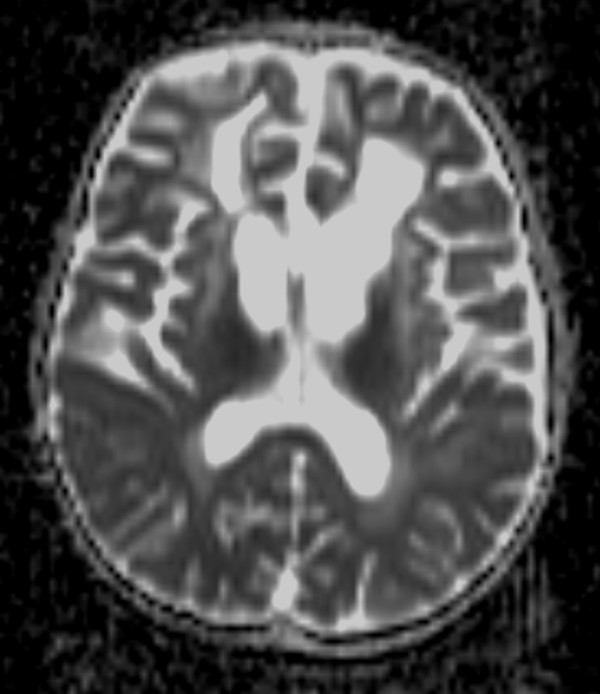
**Magnetic resonance imaging at 5 years, 4 months. a** T2-weighted image shows increase in abnormal signals in the frontal lobe and volume loss in bilateral white matter and deep gray matter. **b** T2-weighted image demonstrates an increase in abnormal signals in the medulla oblongata. **c** Fluid attenuated inversion recovery image shows swelling and high intensity from the medulla oblongata to the top of the cervical spine. **d**, **e** Mild elevation in apparent diffusion coefficient values are recognized in affected areas.

When the patient presented with sudden dyspnea and dysphagia at 5 years 4 months of age, emergency MRI was performed and found a further advance in the following: White matter abnormalities in the frontal lobe, volume loss in bilateral white matter and deep gray matter, and brain stem abnormalities since initial MRI images (Figure 3a, b, c). Mild elevation of apparent diffusion coefficient (ADC) values was noted at affected sites (Figure 3d, e).

## Discussion

Pathological features in Alexander disease include deposits of Rosenthal fibers in astrocytes. Rosenthal fibers contain ubiquitin, heat shock proteins (HSP27) and partially degraded fragments of GFAP. Astrocyte function impairment is considered to cause adverse effects to adjacent structures, such as oligodendroglia and myelin sheath. Some cases result from autosomal recessive inheritance, but the majority is isolated (spontaneous mutations).

MRI is a crucial examination in the diagnosis and observation of Alexander disease. Under the MRI-based diagnostic criteria proposed by van der Knaap [2], at least four of the five imaging findings below must be present for the diagnosis: 1) white matter abnormalities (swelling, signal change, atrophy, cysts) with frontal preponderance, 2) periventricular rim of high signal intensity on T1-weighted images and low signal intensity on T2-weighted images, 3) abnormalities in the basal ganglia and thalami, 4) brain stem abnormalities, 5) contrast enhancement involving one or more of the following structures: ventricular lining, periventricular rim, white matter of the frontal lobes, optic chiasm, fornix, basal ganglia, thalamus, dentate nucleus, and brain stem. In our study, four of the five criteria proposed by van der Knaap except for a periventricular rim were seen at 1 year of age, and MRI findings at 4 years of age showed all five criteria exactly.

High signal intensity on T2-weighted images are reported to correspond to deposits of Rosenthal fibers or associated demyelination, and because these fibers cause breakdown of the blood–brain barrier, contrast enhancement is seen in sites with significant deposits [3,4]. Loss of brain tissue also leads to cyst formation. ADC and MR spectroscopy, although not included in van der Knaap’s diagnostic criteria, have been reported to be useful in diagonosing Alexander disease [4,5]. In our case, elevation of ADC values at the affected areas were recognized (Figure 3d, e), which was thought to correspond with white matter degeneration.

As a result of a nationwide investigation, Yoshida *et al.*[4,5] proposed a new classification on the basis of neurological and MR findings: cerebral Alexander disease (type 1), bulbospinal Alexander disease (type 2), and intermediate form (type 3) [6,7]. According to this classification, our case is classified as cerebral Alexander disease at the onset, and then features of the bulpospinal type become apparent as the disease progresses.

Shiihara *et al.*[7] reported MRI in a long survival case. Unlike our case, cyst formation was not seen in early MRI like in their case. However, in addition to the abnormalities in the frontal lobes, brainstem lesion and brain atrophy became apparent in the progression of the disease in a follow up period of 15 years. Further studies are needed to clarify the relationship between the initial and serial MRI findings and clinical course after diagnosis.

## Conclusion

In our case, the initial MRI and MRI-based serial observations were essential to diagnose and ascertain the image-based progression of clinical symptoms. Initial MRI and MRI-based serial observations were essential to diagnose Alexander disease. Because Alexander disease is a very rare disease with a variety of clinical phenotypes, serial MR studies for long-term survival of infantile cases including our case may be useful for the analysis of the pathophysiological mechanism.

## Consent

Written informed consent was obtained from the mother of the patient for publication of this manuscript and any accompanying images. A copy of the written consent is available for review by the Edior-in-Chief of this journal.

## Competing interests

The authors declare that they have no competing interests.

## Authors’ contribution

FN had contributed to the diagnosis for the MR and CT images of this patient. All authors read and approved the final manuscript.

## References

[B1] Murakami N Tsuchiya T Kanazawa N Tsujino S Nagai T Novel deletion mutation in *GFAP* gene in an infantile form of Alexander Disease Pediatr Neurol 2008 38 50 52 10.1016/j.pediatrneurol.2007.08.017 18054694

[B2] van der Knaap MS Naidu S Breiter SN Blaser S Stroink H Springer S Begeer JC van Coster R Barth PG Thomas NH Valk J Powers JM Alexander Disease: Diagnosis with MR imaging Am J Neuroradiol 2001 22 541 552 11237983 PMC7976831

[B3] Sakakibara T Takahashi Y Fukuda K Inoue T Kurosawa T Nishikubo T Shima M Taoka T Aida N Tsujino S Kanazawa N Yoshioka A A case of infantile Alexander disease diagnosed by magnetic resonance imaging and genetic analysis Brain Dev 2007 29 525 528 10.1016/j.braindev.2007.02.002 17383133

[B4] Yoshida T Nakagawa M Clinical aspects and pathology of Alexander disease, and morphological and functional alteration of astrocytes induced by GFAP mutation Neuropathol 2012 32 440 446 10.1111/j.1440-1789.2011.01268.x 22118268

[B5] Yoshida T Sasaki M Yoshida M Namekawa M Okamoto Y Tsujino S Sasayama H Mizuta I Nakagawa M The Alexander Disease Study Group in Japan Nationwide survey of Alexander disease in Japan and proposed new guidelines for diagnosis J Neurol 2011 258 1998 2008 10.1007/s00415-011-6056-3 21533827

[B6] van der Voorn JP Pouwels PJW Salomoms GS Barkhof F van der Knaap MS Unraveling pathology in juvenile Alexander disease: serial quantitative MR imaging and spectroscopy of white matter Neuroradiol 2009 51 669 675 10.1007/s00234-009-0540-9 PMC2744817 19484233

[B7] Shiihara T Yoneda T Mizuta I Yoshida T Nakagawa M Shimizu N Serial MRI changes in a patient with infantile Alexander disease and prolonged survival Brain Dev 2011 33 604 607 10.1016/j.braindev.2010.10.007 21041050

